# Using natural language processing and machine learning to identify breast cancer local recurrence

**DOI:** 10.1186/s12859-018-2466-x

**Published:** 2018-12-28

**Authors:** Zexian Zeng, Sasa Espino, Ankita Roy, Xiaoyu Li, Seema A. Khan, Susan E. Clare, Xia Jiang, Richard Neapolitan, Yuan Luo

**Affiliations:** 1 Department of Preventive Medicine, Feinberg School of Medicine, Northwestern University, Chicago, IL USA; 2 Department of Surgery, Feinberg School of Medicine, Northwestern University, Chicago, IL USA; 3 Department of Social and Behavioral Sciences, Harvard T.H. Chan School of Public Health, Boston, MA USA; 4 Department of Biomedical Informatics, University of Pittsburgh, Pittsburgh, PA USA

**Keywords:** Breast cancer local recurrence, EHR, NLP, SVM

## Abstract

**Background:**

Identifying local recurrences in breast cancer from patient data sets is important for clinical research and practice. Developing a model using natural language processing and machine learning to identify local recurrences in breast cancer patients can reduce the time-consuming work of a manual chart review.

**Methods:**

We design a novel concept-based filter and a prediction model to detect local recurrences using EHRs. In the training dataset, we manually review a development corpus of 50 progress notes and extract partial sentences that indicate breast cancer local recurrence. We process these partial sentences to obtain a set of Unified Medical Language System (UMLS) concepts using MetaMap, and we call it positive concept set. We apply MetaMap on patients’ progress notes and retain only the concepts that fall within the positive concept set. These features combined with the number of pathology reports recorded for each patient are used to train a support vector machine to identify local recurrences.

**Results:**

We compared our model with three baseline classifiers using either full MetaMap concepts, filtered MetaMap concepts, or bag of words. Our model achieved the best AUC (0.93 in cross-validation, 0.87 in held-out testing).

**Conclusions:**

Compared to a labor-intensive chart review, our model provides an automated way to identify breast cancer local recurrences. We expect that by minimally adapting the positive concept set, this study has the potential to be replicated at other institutions with a moderately sized training dataset.

**Electronic supplementary material:**

The online version of this article (10.1186/s12859-018-2466-x) contains supplementary material, which is available to authorized users.

## Background

Breast cancer is one of the most prevalent cancers amongst women. In order to improve breast cancer outcomes, many research groups have focused on developing new treatment strategies [1, 2], identifying new biomarkers [3], and studying related risk factors [4–8]. Carrying out these studies requires a direct and effective outcome measure. Local recurrences in breast cancer, or ipsilateral recurrences, refer to cases in which the malignancy occurs at the original site after a lumpectomy or in the chest wall area after mastectomy. When evaluating local therapies, such as radiation or breast conservation surgery, local recurrence-free survival is an efficacious endpoint for outcome measurement [9].

There have been significant developments in maintaining electronic health records (EHRs) within the last decade, which has made clinical data increasingly available in electronic form. Compared to prospective studies, the abundant data extracted from EHRs is an attractive resource for retrospective research, such as low-cost case-control studies. This resource has allowed researchers to conduct large cohort studies to answer various clinical questions. Furthermore, biopsies and tumors stored in biobanks can be linked to the EHR by matching patient identifiers, which makes it possible to study genetic and phenotypic patterns simultaneously [10–12].

Clinical data such as signs and symptoms, and disease status and severity are often recorded in narratives in EHRs. For example, progress notes are a record of events during a patient’s office visit or hospitalization that communicate opinions, findings, and plans between healthcare professionals. A well-documented progress note is complete, accurate, and concise for the care delivered, including diagnosis and treatments [13]. To identify recurrences from the narratives, researchers still heavily rely on manual chart review. In addition to being error-prone, the review process is both labor-intensive and time-consuming, making it difficult to scale to large cohort studies. The abundance of information in the free text makes natural language processing (NLP) an indispensable tool for text-mining [14, 15]. Various NLP tools have been developed to extract features from free text for patient profile representation. Such features can further be used for cohort classification or clustering. Numerous studies have applied NLP to extract meaningful information from clinical narratives, such as identification of disease status [16–21], drug-drug interactions [22], and adverse drug events [23].

To identify breast cancer local recurrence from EHRs, Lamont et al. used ICD9 codes as features. They obtained area under receiver operating characteristic curve (AUC) scores of 0.84 and 0.97 for two-year censoring and five-year censoring periods, respectively [24]. However, claims data have limited validity in inferring cancer recurrences due to its low accuracy [25]. Strauss et al. used morphology codes and anatomic sites to detect breast cancer local recurrence [26]. They achieved a positive predictive value (PPV) score and a negative predictive value (NPV) score of 0.94 and 1, respectively. Nevertheless, the method required that the pathology report be well-documented in a standard format. In reality, the reports are written in different formats across institutions and require special care for NLP systems to unify the cross-institutional variations [18, 19]. Haque et al. used a hybrid of pathology reports and EHR data [27]; yet the model resulted in a low precision of 65.6%. More recently, Carrell et al. proposed a method to combine pathology reports, radiation notes, clinical reports, and EHR data to detect recurrences [28]. The authors achieved an F-measure score of 0.84 in the training set and 0.72 in the test set. Furthermore, the system was not able to distinguish between local recurrences and distant recurrences.

The National Program of Cancer Registries (NPCR) was launched to capture cancer patient information. One of its major tasks is to capture a comprehensive history, diagnosis, treatment, and disease status for each cancer patient. However, breast cancer local recurrence information is often not well documented as structured data at individual hospitals and medical institutions [29, 30]. In addition, a patient may have risks of developing such an event for an extended period in his or her lifetime [31, 32]. The magnitude of work needed to capture and maintain pathophysiologic data for NPCR is not trivial [33]. In contrast, progress notes serve as a tool to communicate opinions, findings, and plans between healthcare professionals. Using the detailed information in progress notes, a well-designed NLP algorithm should be able to accurately identify local recurrences.

Motivated by the limitations of previous studies, we aimed to develop a tool to identify breast cancer local recurrences. Such a model should be able to be built and replicated without intensive labor input. The input data for the model should be easily obtainable, such as progress notes and number of pathology reports. Furthermore, the model should be able to identify breast cancer local recurrences accurately. With these aims, in this study we applied support vector machine (SVM) to quantitatively assess the likelihood of a patient having a local recurrence, using a hybrid of narratives in progress notes and the number of breast cancer surgical pathology reports that were generated at least 120 days following the date of primary diagnosis.

## Methods

**Fig. 1:**
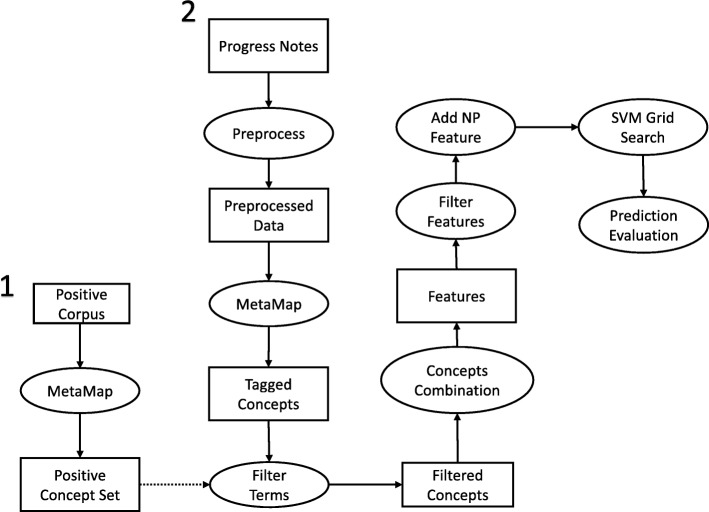
Diagram of the workflow of the study. Processing steps are in the circles; narratives, concepts, and features are in the squares. NP represents the number of pathology reports generated at least 120 days after the first primary diagnosis. We start with pipeline 1 by manually going through a development corpus of 50 randomly selected positive progress notes to build a positive concept set. We then start pipeline 2 by going through every patient’s progress notes. The dash line indicates that only concepts falling in the positive concept set are retained

An overview of the workflow employed in this study is shown in Fig. 1. We start by first defining a positive concept set, which is marked as pipeline 1 in Fig. 1. In the training dataset, we manually review a development corpus of 50 randomly selected progress notes and extract partial sentences that indicate breast cancer local recurrence. We process these partial sentences to obtain a positive set of concepts using MetaMap. The work in pipeline 1 is described in the “Positive concept set” section. After obtaining the positive concept set, we start pipeline 2 as marked in Fig. 1. We first preprocess the narratives as described in the “Data preprocessing” section. We then apply MetaMap to annotate relevant medical events in the preprocessed notes. Only concepts that fall within the positive concept set are retained. Different concepts extracted from the same sentence are combined to generate power sets as additional features. The process to generate features from narratives are presented in the “Feature generation” section. In addition, the number of pathology reports generated at least 120 days following the date of primary diagnosis is used as a feature. The intuition and method of generating such a feature is described in the “Number of pathology report” section. These features together are used to train a support vector machine that identifies breast cancer local recurrences. After training and tuning the model, we rigorously evaluate the model’s performance in local recurrence prediction. The details of training and evaluating the model are presented in the “Prediction model” and “Model evaluation” sections, respectively.

### Positive concept set

The first step in this study is to build a positive concept set. As shown in Table 1, examples of positive and negative partial sentences are extracted from progress notes. In the positive examples, we can infer the local recurrences by reading the partial sentences. The word ‘recurrence’ or ‘recurrent’ appears in most cases. However, among the negative examples, the word ‘recurrence’ or ‘recurrent’ alone is not informative enough for us to infer breast cancer local recurrences. In fact, we need additional words to infer the local recurrences correctly. To build a comprehensive positive concept set for recurrence inference, we manually go through a development corpus of 50 randomly selected positive progress notes and extract partial sentences that are associated with breast cancer local recurrence. These extracted partial sentences are summarized in Additional file 1: Table S1. To illustrate, one of the partial sentences identified in this study is as follows: ‘after her chest wall breast cancer recurrence excision’. This partial sentence indicates that the patient has a chest wall recurrence and also had an excision. In total, 93 partial sentences are identified from the 50 progress notes. These partial sentences are tagged by MetaMap, which is an NLP application to map the biomedical text to the Unified Medical Language System (UMLS) Meta-thesaurus [34]. The concept unique identifier (CUI) corresponding to each concept is obtained. To reduce noise, CUIs that are not related to breast cancer local recurrence are manually filtered. CUIs for words such as ‘then’, ‘the’, ‘to’, etc. are filtered and discarded. Finally, 48 CUIs are retained; they are listed in Additional file 2: Table S2. These CUIs together represent a set of positive concepts that describe breast cancer local recurrence. To illustrate, the partial sentence ‘history of recurrent breast cancer’ contributes three CUIs, namely ‘history of malignant neoplasm of breast’ (C1997028), ‘Personal history of primary malignant neoplasm of breast’ (C1387407), and ‘Recurrent’ (C2945760), to the positive concept set.

**Table 1:** Positive and negative examples of partial sentences indicating local recurrences

	Partial sentences
Positive Examples	Now with newly diagnosed DCIS recurrence
	She initially received breast-conserving therapy with radiotherapy for a right breast cancer in 1990 and now she presents with a right-sided pT1b N0 (SN) infiltrating lobular carcinoma.
	She was found to have an ipsilateral breast tumor recurrence
	Female with a history of left breast stage IIIC infiltrating ductal carcinoma who was treated with breast conserving surgery and adjuvant chemo radiation in 2010 who was recently diagnosed with a cancer in the ipsilateral breast
	Is currently receiving chemotherapy for her recurrent breast cancer
Negative Examples	We recommended the patient undergo adjuvant radiation therapy with the goal of decreasing local regional recurrence and possibly increasing the overall long-term survival
	Carefully explained to her that removing the right breast would not change her overall survival and had minimal impact on her recurrence
	She presents with recurrence of depressive symptoms associated with new breast cancer diagnosis
	Pt very concerned and anxious as some of her friends have been diagnosed with recurrent breast cancer.
	Despite this stressor, and the attendant emotional strain, she appears to be coping well at present and is dealing well with fear of recurrence and medical issues

### Data preprocessing

A number of preprocessing steps are performed on the progress notes. We remove duplicate copies, divide the notes into sentences, and remove non-alphanumeric symbols. Following these preprocessing steps, we annotate the medical concepts in the sentences using MetaMap. The surrounding semantic context is determined. If multiple CUIs are mapped, the one with maximum MMI score (a score ranked by MetaMap) is retained. CUIs that are tagged as negated by NegEx [35] are excluded (NegEx is a negation tool configured in MetaMap). In order to completely and accurately exclude negations or unrelated contextual cues, such as a differential diagnosis events, sentences with negation contextual features (e.g. contain keyword of ‘no’, ‘rule out’, ‘deny’, ‘unremarkable’) and uncertain contextual features (e.g. contain keyword ‘risk’, ‘concern’, ‘worry’, ‘evaluation’) are also removed, where the customized list of contextual features are obtained from the development corpus. Following the filtering, CUIs that fall outside the positive concept set are excluded.

### Feature generation

After data preprocessing and concept mapping, CUIs that fall within the positive concept set are used as features to train our model. However, a single CUI sometimes is not informative enough for us to accurately infer a local recurrence. In the previous example, using the CUI ‘history of malignant neoplasm of breast’ (C1997028) or ‘recurrent’ (C2945760) alone, we could not infer a breast cancer local recurrence. Nevertheless, we can properly infer a breast cancer local recurrence using these two CUIs together. Driven by this observation, we generate power sets as additional features using a combination of any two and three CUIs that are extracted from the same sentence. Using the above example of ‘history of recurrent breast cancer’ for illustration, nine features (C1997028), (C1387407), (C2945760), (C1997028, C1387407), (C1997028, C2945760), (C1387407, C2945760), (C1997028, C1387407, C2945760) are generated.

**Fig. 2:**
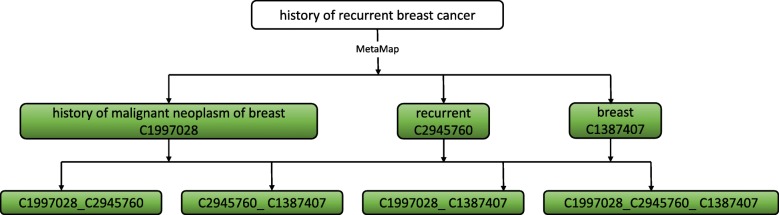
Constructing features using one partial sentence. Green circles are generated features for the model

Figure 2 shows this example to illustrate the processes used to generate power sets. Note that we have used power set to refer to the conventional power set excluding the empty set.

To identify recurrences more precisely, additional features such as the number of pathology reports can be assessed. The intuition and reasoning for implementing such a feature are that patients with local recurrences tend to have more pathology reports in their records because a pathology report is required for breast cancer recurrence diagnosis in most situations. Most of the diagnosed women get a core needle biopsy and have a high probability of undergoing surgery. Pathology reports are generated for these events. Therefore, women who had a recurrence will ordinarily have more pathology reports generated in their EHRs, which means the number of pathology reports is associated with local recurrence. In this study, the number of pathology reports generated at least 120 days after the first primary diagnosis is obtained as an additional feature. This number is obtained from the Northwestern Medicine Enterprise Data Warehouse (NMEDW).

### Data description

The NMEDW is a joint initiative across the Northwestern University Feinberg School of Medicine and Northwestern Memorial HealthCare. The NMEDW is used to retrieve data. Patients diagnosed with breast cancer at Northwestern Memorial Hospital between 2001 and 2015 are identified by ICD9 codes and are included in the study. In total, 6899 subjects are identified and included in the model training and evaluation. These 6899 subjects’ notes are divided and manually annotated by a postdoc fellow (co-author XL), and a Ph.D. student (co-author ZZ) over 15 months. In total, 569 (8.25%) local recurrences are identified among the 6899 subjects in this round of manual curation.

The five-year local recurrence rate is 8.8% for ER-positive breast cancer and is 6.9% for ER-negative breast cancer [32], and often only a small amount of recurrences are identified in most study cohorts. Without a large cohort, models trained using such a dataset may experience problems with generalizability. In the previous studies, only 12 local recurrence patients were identified by Lamont et al., and 32 local recurrence patients were identified by Strauss et al. [24, 26]. Our goal is to build a much larger cohort. As a first step, one medical informatics student (co-author ZZ) and one public health student (co-author XL) annotate the 6899 patients and randomly select 201 subjects with local recurrence and 500 subjects without local recurrences. These 701 subjects’ records are further annotated by a medical student (co-author AR), and a breast surgery fellow (co-author SE), thus forming a double-annotation of 701 subjects (a record is annotated by ZZ/XL and AR/SE). We intentionally keep the division of 701 subjects between ZZ and XL different from the division between AR and SE for maximal cross check. The items without agreement are resolved by discussion between the two annotators. In the second round of annotation, we identify 16 subjects who do not have a local recurrence within the 201 subjects who were previously identified with local recurrences, and we identify 8 local recurrences within the 500 subjects, who were previously identified without local recurrences. After the second round of annotation and confirmation, of the 701 subjects, 193 subjects are identified as having a local recurrence and 508 subjects are identified as having no local recurrence. In the second round of annotation, we identify 24 misclassified notes out of 701 samples and the error rate is 3.4%. The Cohen’s kappa score for the double annotation (ZZ/XL vs. AR/SE) is 0.92. With this high accuracy and high kappa score (0.81–1 kappa considered as almost perfect agreement [36]), the first round annotation outside the 701 samples is a reasonable “silver standard” for evaluating the generalization performance of our model. Among the study cohorts, 701 samples are double-annotated and 6198 samples are single-annotated. In total, 561 (8.13%) subjects developed a breast or chest wall local recurrence. The number is close to what appears in literature [32]. The cohort distributions appear in Table 2.

**Table 2:** Training cohort and test cohort distributions

	Total	Local Recurrence	Percentage (%)	Overall percentage (%)
Double annotation set	701	193	27.53%	8.13%
Cross-validation set	490	143	29.18%	
Held-out test set	211	50	23.70%	
Single annotation set	6198	368	5.94%	

### Model evaluation

We utilized the support vector machine (SVM) because of its widely-acknowledged generalizability. Before training the model, a chi-square test is applied to select features to obtain a reasonable feature sample ratio. To include only features that are highly associated with outcomes, only the top 50% of features are retained for subsequent modeling. After filtering the features, we perform a five-fold cross-validation using the training data to tune the model’s parameters. Parameters of gamma and C for the model are selected by a grid search with C ranging from 1 to 100 with interval spacing equal to 10, and gamma ranging from 0.0001 to 0.01 with interval spacing equal to 0.001. Kernel types ‘radial basis function’, ‘linear’, ‘poly’, and ‘sigmoid’ are tested. The parameters with the best performance using the micro-averaged F1 score in the cross-validation are retained. Cross-validation favors a radial basis function kernel for all the settings in our experiments.

The 701 double-annotated subjects are randomly split into a training set and a held-out test set according to a 7:3 ratio. In our experiments, we train three baseline classifiers using various feature configuration. The three baselines are: uses a full set of medical concepts tagged by MetaMap, uses only the filtered features generated from progress notes without the number of pathology reports, and uses the bag of words as features. The function of TfIDFVectorizer in scikit-learn was used to convert the raw documents to a matrix of TF-IDF features for bag of words.

## Results

**Fig. 3:**
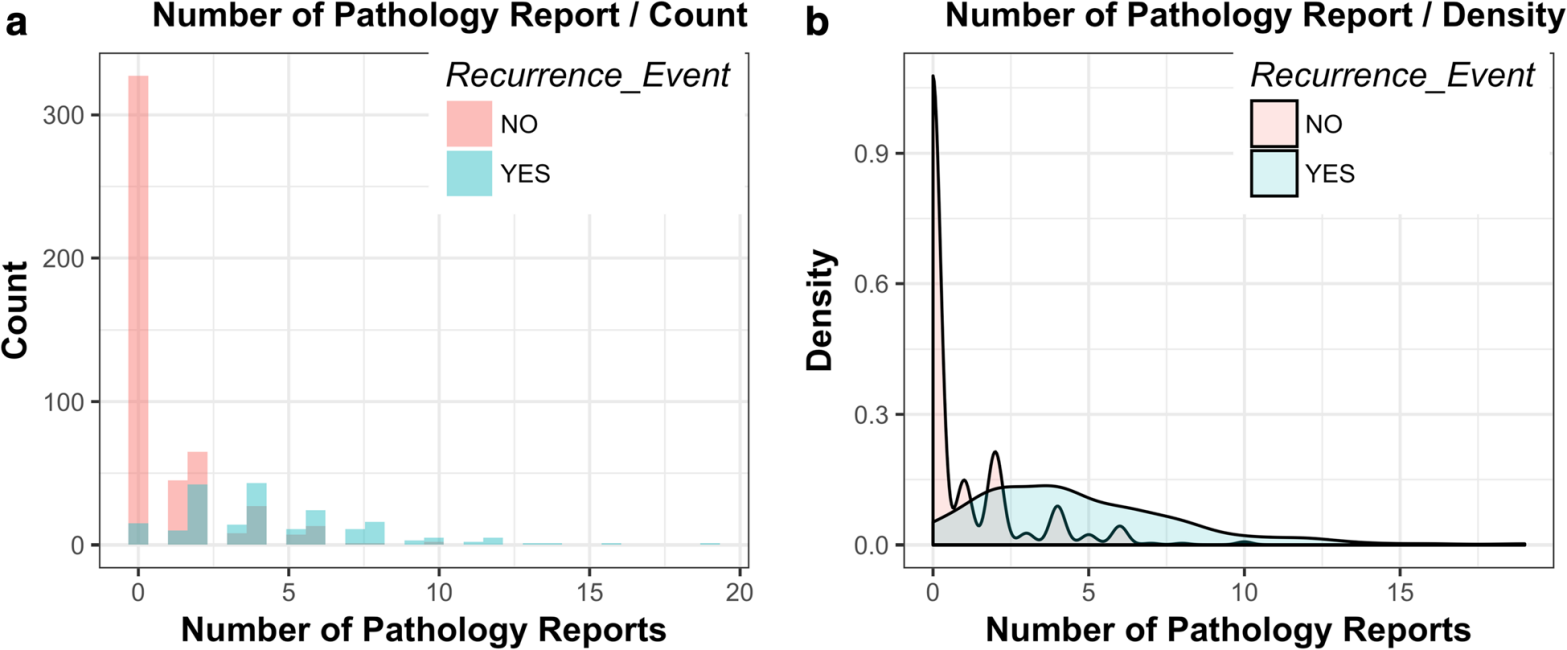
Histogram plot for number of pathology reports. **a** frequency distribution of subjects with and without local recurrence; **b** density distribution of subjects with and without local recurrence

Among the 701 patients in the training set, the average number of pathology reports is 1.98 (95% CI =±0.16). Among the 193 patients with a local recurrence, the average number of pathology reports is 4.55 (95% CI = ±0.44). Among the 508 patients without a local recurrence, the average number of pathology reports is 0.92 (95% CI = ±0.15). A histogram and a density plot for the number of pathology reports stratified by subjects with and without local recurrence appear in Fig. 3.

Each of the 701 subjects was annotated by two annotators and correctly labeled as either having breast cancer with a local recurrence or without a local recurrence. We modeled the task as a classification problem and also reported the probability. In total, 4151 features were generated in our proposed model. A total of 17,897, 4150, and 57,612 features were generated for baselines ‘full MetaMap’, ‘filtered MetaMap’, and ‘bag of words’, respectively. Table 3 shows the evaluation results of our model in comparison to the other three methods in the cross-validation. The evaluation metrics include precision, recall, F-measure, and AUC. It is clear that full MetaMap features outperformed the bag of words. We compared our model with full MetaMap and filtered MetaMap using the Student’s t-test. The evaluation metrics with significant changes (*p* < 0.05) in Table 3 are marked. We see improvements on AUC, recall, and F-measure in our model compared to full MetaMap or filtered MetaMap. In the cross-validation, the AUC for our model is 0.93 with a standard deviation of 0.01.

**Table 3:** Cross-validation results using different methods

Methods	P (SD)	R (SD)	F (SD)	AUC (SD)
Filtered MetaMap +Pathology Report Count (4151)	0.84 (0.04)	**0.76 (0.02)**	**0.80 (0.02)**	0.93 (0.01)
Full MetaMap (17897)	0.80 (0.06)	0.48 (0.05)	0.60 (0.05)	0.83 (0.03)
Filtered MetaMap (4150)	0.82 (0.03)	0.67 (0.02)	0.74 (0.02)	0.90 (0.01)
Bag of Words (57612)	0.69 (0.07)	0.42 (0.062)	0.52 (0.06)	0.78 (0.03)

The number in the parenthesis in the first column is the number of features. The number in parenthesis in the 2nd~5th columns is standard deviation

Gray shade indicates baseline methods

*P* stands for precision, *R* stands for recall, *F* stands for f score, *AUC* stands for area under the receiver operator characteristic curve, and *SD* is standard deviation

Table 4 shows the evaluation results of the model for classification in comparison with the three other methods in the held-out test. The AUC score is 0.87 for our proposed model, which outperformed the other methods and is consistent with the cross-validation result.

**Table 4:** Held-out test results using different methods

Methods	P	R	F	AUC
Filtered MetaMap +Pathology Report Count (4151)	0.74	0.84	0.79	0.87
Full MetaMap (17897)	0.66	0.34	0.45	0.80
Filtered MetaMap (4150)	0.71	0.78	0.74	0.84
Bag of Words (57612)	0.53	0.43	0.48	0.74

The number in the parenthesis in first column is the number of features

Gray shade indicates baseline methods

*P* stands for precision, *R* stands for recall, *F* stands for f score, *AUC* stands for area under the receiver operator characteristic curve

We used the 701 double annotated data to fit an SVM model. The fitted model was then used to predict labels on the rest of 6198 samples. The predictions were compared with the annotated labels, and we obtained a precision of 0.50, a recall of 0.81, an f-measure of 0.62, and an AUC score of 0.87.

Using the training data, we performed a feature study. We extracted the coefficient for each feature from a fitted linear SVM model. The top 10 ranked features appear in Table 5. The UMLS concept preferred name is also presented in the table. Two power sets were ranked among the top 10 features, which suggests that the power sets have a significant role in predicting the right label. Of note, the number of pathology reports ranked as the 101st feature with a coefficient score equal to 0.12.

**Table 5:** The top-ranked features with the corresponding coefficient in the model and the UMLS concept preferred name for the CUIs

Feature	Coefficient	UMLS Concept Preferred Name
C0278493	0.66	‘Recurrent breast cancer’
{C0007124; C0222600; C0222600}	0.46	{‘Noninfiltrating Intraductal Carcinoma’; ‘Right breast’; ‘Right breast’}
C0920420	0.43	‘Cancer recurrence’
C1458156	0.41	‘Recurrent Malignant Neoplasm’
C2945760	0.40	‘Recurrent’
C0235653	−0.36	‘Malignant neoplasm of female breast’
C0277556	0.36	‘Recurrent disease’
C1512083	− 0.35	‘Ductal’
{C0007124; C0205090; C0262512}	0.32	{‘Noninfiltrating Intraductal Carcinoma’; Right; ‘History of present illness’}
C4042789	0.30	‘Right-Sided Breast Neoplasms’

## Discussion

In the study, we used MetaMap to identify words or phrases in the text and map them to the Unified Medical Language System (UMLS). The UMLS mapping has the benefit of increasing semantic interoperability. Our proposed model was able to retrieve breast cancer local recurrence using the combination of UMLS concepts and the additional feature of pathology report counts. In the study, we obtained better performance using Full MetaMap concepts compared to bag of words. This improvement is mainly due to the UMLS mapping. Before training the model, we performed a feature selection. Some of the redundant CUIs appear in almost every patients’ notes, and some CUIs appear only in one or two patients’ notes. This feature selection process can remove these redundant or unrelated features, yielding a reasonable feature and sample ratio. The AUC score of our proposed model was 0.93 (±0.01) in a cross-validation and was 0.87 in a held-out test. The AUC score of our model outperformed the model proposed by Lamont et al. [24] in a five-year censoring period. In addition, we have a larger sample size in this study. Compared to the hybrid model proposed by Haque et al. [27], our model achieved a higher precision and takes less effort to replicate.

To date, most of the tools used to retrieve breast cancer local recurrence are rule-based systems using pathology reports [26, 27]. However, different institutions may have different clinical documentation systems and styles, which poses challenges in generalizing the work to multiple institutions. In this study, we dismissed the requirement of documentation formats by utilizing the narrative text from progress notes and the number of pathology reports. In particular, we use the number of pathology reports without applying our NLP pipeline. Pathology reports are usually heavily templated in different institutions using different templates [37]. In the future, we plan to develop a processing component in our NLP pipeline and integrate pathology report text from multiple institutions to explore the impact of varying pathology report templates to generalizability.

Using the reported probability, if we use 0.5 as a cutoff, we obtained 41 false negatives and 32 false positives. The 41 false negatives have an average of 2.6 pathology reports per patient, which is significantly (*p*-value =5.12E-06) lower than 5.11 in the 152 true positives. Among these 41 false negatives, the number of pathology number is highly correlated (Pearson correlation coefficient = 0.69) with the prediction probability. Within the 41 false negatives, we examined 14 cases with more than two pathology reports but have low prediction probability smaller than 0.2. A majority of them have a small number of progress notes available in the database. Thus, they have minimal breast cancer recurrence related information recorded in their notes. For the 32 false positives, most cases were misidentified because these women’s contralateral occurrences or distant recurrences were incorrectly categorized as a local recurrence. Contralateral breast cancer is defined as breast cancer (invasive or DCIS) that develops in the opposite breast after the detection of primary breast cancer. Distant recurrence is defined as cancer spreading to other organs. For example, one woman had a contralateral event and a distant recurrence event. In one of her progress notes, the following was recorded: “History of Present Illness: 50 y/o female with h/o recurrent breast CA in chemo who presents with fever”. In this situation, the ‘recurrent breast cancer’ refers to the distant recurrence. Furthermore, the patient has two pathology reports at least 120 days after the primary diagnosis for her contralateral event. Without knowing the site information about her recurrent cancer, the algorithm could not distinguish whether or not this was a local recurrence. Within these 32 false positives, several cases had a high prediction probability because they had a high number of pathology report counts. However, the high number of pathology report counts were generated because the patients visited the hospital multiple times for benign biopsies or re-excision.

In summary, the sentences in progress notes contain rich information. In addition, the number of pathology reports is easy to retrieve. This added feature significantly improved the model’s performance (*p*-value comparing recalls: 0.001; p-value comparing F-measures: 0.004). In particular, recall significantly improved while precision was maintained or improved. Using these features, we can accurately identify breast cancer local recurrences. For generalizability purpose, part-of-speech, format, or style information were not included for model training. Since our method is not sensitive to the particular format of progress notes and pathology reports, this study is easy to replicate and can reduce the time-consuming work required to manually identify local recurrences. In the future, structured data such as biomarkers, lymph node status, tumor characteristics, etc. will be included and evaluated to see if it can improve the model’s performance.

However, the model still has several limitations. Replicating the model requires building a set of positive concepts. Without having a known positive event set, it is difficult to build a filter. To address this concern, we released the positive concept set for local recurrences in the supplementary material. The model also has another limitation in that it can confuse local recurrence with distant recurrence, especially when the patient also has a contralateral event. The mention of ‘recurrent breast cancer’ for distant recurrence and a high pathology number for a contralateral event together can lead to an incorrect prediction. We also acknowledge that this is a skewed data set because the event rate is low. To address this problem, we performed the analysis constructing and using a less skewed case-control data set. The statistics of Tables 3 and 4 reflect the enrichment of the training corpus with local recurrence cases, which has been artificially inflated in the gold standard dataset from the true value of 7–9% in the population [32] to 28%. We are aware that the precision is influenced by incidence, so we further validated the model by reporting the model performances using the 701 double annotated samples (gold standard dataset) plus the 6198 single annotated samples (silver standard dataset), which together have an incidence rate matching the population rate. The generalization test showed similar performance and our future goal is to double annotate the entire cohort and release the double annotated dataset for secondary research use. Limitations also include not analyzing pathology report text and not considering richer semantic relations between concepts due to generalizability considerations. We plan to address those issues and continue to improve our model. In this study, position information (where the feature appears in a sentence) of the CUI feature are not included in our model. This information can possibly help remove some redundant features and increase the model performance. In the future, instead of using position-sensitive power set, we plan to use graph-based representation to capture the relations between CUIs with more accuracy [38].

## Conclusions

Our aim was to utilize narrative sentences to generate medical concepts, mine relevant concepts, and combine the mined concepts for feature generation. We applied NLP to breast cancer local recurrences identification by integrating a flexible number of medical concepts, their power sets, and the additional feature of the number of pathology reports. We then applied support vector machines (SVMs) to identify local recurrences in breast cancer patients. Our evaluation shows that the classifier significantly outperforms three baseline models using either full MetaMap concepts, filtered MetaMap concepts, or bag of words as features. We expect that by minimally adapting the positive concept set (48 CUIs in our study), this study has the potential to be replicated at other institutions with a moderate sized training dataset (701 samples in our study). Further development of this model will allow more accurate data-mining and significantly less time-consuming manual chart review. This is particularly relevant in an era where evidence-based medicine is increasingly scrutinized and there is a growing interest in data-driven discoveries.

## Additional files

Additional file 1: **Table S1.** Positive sentences. We went through a development corpus of 50 randomly selected positive progress notes and extracted partial sentences that indicated a breast cancer local recurrence event. (XLS 27 kb)

Additional file 2: **Table S2.** Positive CUIS. A dictionary of CUISs and the corresponding CUI preferred name that were retained after filtering. (XLS 23 kb)

## Abbreviations

AUC: Area under receiver operating characteristic curve
CUI: Concept unique identifier
EHRs: Electronic health records
NLP: Natural language processing
NPCR: National Program of Cancer Registries
NPV: Negative predictive value
PPV: Positive predictive value
SVM: Support vector machine
UMLS: Unified Medical Language System
